# Decoding pain’s neural rhythm: Gamma oscillation mechanisms, therapeutic modulation, and translational challenges in pain management

**DOI:** 10.3389/fnins.2025.1656588

**Published:** 2025-09-18

**Authors:** Chen Zhang, Lanze Xiao, Min Xiao, Xu Zhang, Chengpeng Zhang, Bangjiang Fang, Hongwu Tao

**Affiliations:** ^1^Department of Emergency, Hubei Provincial Hospital of Integrated Chinese and Western Medicine (Xinhua Hospital Affiliated to Hubei University of Chinese Medicine), Wuhan, China; ^2^College of Clinical Chinese Medicine, Hubei University of Chinese Medicine, Wuhan, China; ^3^Department of Rehabilitation, Yichang Hospital of Traditional Chinese Medicine, Yichang, China; ^4^Department of Emergency, Longhua Hospital, Shanghai University of Traditional Chinese Medicine, Shanghai, China; ^5^The Second Affiliated Hospital of Liaoning University of Traditional Chinese Medicine, Shenyang, China

**Keywords:** pain, gamma oscillations, neural oscillation, cerebral cortex, therapeutic modulation

## Abstract

Gamma oscillations (30–100 Hz), as a rhythmic neuronal activity within the central nervous system, play a pivotal role in the initiation, progression, and therapeutic management of pain. By synthesizing relevant experimental and clinical evidence, this review examines pain-induced alterations in gamma oscillations across cortical regions and surveys recent gamma oscillation-based therapeutic interventions for pain management. Gamma oscillations in key cortical areas—including the somatosensory cortices, prefrontal cortex, anterior cingulate cortex, orbitofrontal cortex, and insula—are significantly modulated by pain. Therapeutic approaches encompass pharmacological agents (e.g., morphine, ketamine) and non-pharmacological modalities (e.g., electroacupuncture, transcutaneous electrical nerve stimulation). Emerging therapies such as virtual reality and music-based analgesia offer novel mechanistic insights. However, current research faces limitations, including prevalent insufficient sample sizes. Future research should leverage AI to conduct real-world studies, establish electroencephalogram databases, and investigate the role of gamma oscillations in disease pathology. This will advance precision pain management and optimize therapeutic outcomes for patients.

## 1 Introduction

Pain, as an evolutionarily conserved defense alarm, is ubiquitous in daily life and particularly prevalent in medical practice. It induces multifaceted impairments encompassing both physical and emotional domains, such as cognitive dysfunction, depression, sleep disturbances, social disabilities, and diminished motor skills (Antunes et al., 2024). The International Association for the Study of Pain currently defines pain as ‘an unpleasant sensory and emotional experience associated with, or resembling that associated with, actual or potential tissue damage (Raja et al., 2020). Because the perception and management of pain rely on the higher neural centers, research predominantly focuses on alterations in the central nervous system. Neural oscillations represent rhythmic or repetitive neuronal activities within this system, generated through diverse mechanisms primarily driven by interactions between individual neurons or neuronal networks (Kim and Davis, 2020). Accumulating clinical evidence demonstrates that distinct oscillatory patterns and their inter-regional synchrony play pivotal roles in pain processing (Tan et al., 2021), such as facilitating the integration and encoding of nociceptive information.

Pain-related neural oscillations span a broad frequency spectrum, ranging from infraslow fluctuations (<0.1 Hz) through theta, alpha, and beta bands up to gamma band oscillations (GBOs; Ploner et al., 2017). GBOs (30–100 Hz) are ubiquitously distributed throughout the brain, endowing neurons with millisecond temporal precision (Kim and Davis, 2020). These oscillations integrate features of sensory stimuli by generating synchrony across discrete cortical regions (Whittington et al., 1998). The generation of GBO primarily involves interactions between gamma-aminobutyric acid-ergic (GABAergic) parvalbumin-positive (PV) interneurons, or between PV interneurons and pyramidal cells (Buzsáki and Wang, 2012), with their frequency and patterning being stringently governed by GABAergic kinetics (Traub et al., 1996).

The association between GBO activity and pain has been robustly evidenced across diverse conditions, including but not limited to: short-term acute pain (Zhou et al., 2018; Tiemann et al., 2010; Liu et al., 2015), persistent pain (Schulz et al., 2015; Peng et al., 2014), chronic pain (Cardoso-Cruz et al., 2013), as well as pain sensitivity (Hsiao et al., 2020) and tolerance (Zhou et al., 2020), etc., as summarized in Table 1.

**Table 1:** The association between GBO activity and pain has been robustly evidenced across diverse conditions.

Pain characteristics	GBO dynamics
Tonic Pain	A positive relationship between pain intensity and GBOs.
Ongoing pain	A positive association between ongoing pain intensity and prefrontal GBOs.
Chronic Inflammatory Pain	The increased gamma activity occurred mainly at electrodes over primary somatosensory cortices.
Chronic neuropathic pain	Enhanced gamma activity located mainly over the prefrontal cortex (PFC) and cerebellar areas, the enhanced gamma power was positively correlated with pain intensity.
Generalized hyperalgesia	Enhancement in high gamma oscillatory activities in the anterior cngulate cortex (ACC) has been shown to be important and likely play a causal role.
Pain sensitivity	the high pain sensitivityT was inversely correlated with the relative power of GBOs in the bilateral insula, posterior cingulate cortex (PCC), and primary motor cortex regions.
Acute pain	The power at gamma frequency band increased.
Brief thermo-nociceptive	Brief thermo-nociceptive stimuli elicit high-frequency GBOs in the insula.

Significant findings have emerged regarding the attributes of GBO in pain processing. Specifically, GBO amplitude demonstrates a close correlation with perceived pain intensity (Gross et al., 2007) and reliably predicts interindividual variations in pain sensitivity (Hsiao et al., 2020). Through time-frequency analysis of electroencephalogram (EEG) signals, Lyu et al. identified two distinct consecutive GBO components implicated in pain processing. The early-latency GBO amplitude showed positive correlations with both intensity and unpleasantness ratings, while the emotion-modulation effect (negative vs. positive contexts) of late-latency GBO amplitude positively correlated with pain unpleasantness (Lyu et al., 2022).

However, the effects of GBO are broad and not limited to pain. GBOs have been extensively recorded across broad cortical and subcortical regions, including but not limited to visual, auditory, somatosensory, motor (Ichim et al., 2024) and olfactory cortices, thereby playing roles in accurately perceiving the types of odors (Yang et al., 2022), the successful formation and retrieval of episodic memory (Griffiths and Jensen, 2023), and the fine calibration of motor movements, etc. (Tamás et al., 2018). They also have feedback effects on stimuli such as vision, hearing, and attention, and can be modulated by auditory (Schadow et al., 2007), visual (Holt et al., 2024), attentional (Tiesinga and Sejnowski, 2009), and even olfactory stimuli (Neville and Haberly, 2003). Their effects include, but are not limited to: A distinct dependence between sound intensity and GBOs (Schadow et al., 2007). Enhanced oscillatory power driven by auditory beat stimuli (Anjos et al., 2024). Strong and locally synchronized 20–70 Hz oscillatory responses triggered by visual stimuli (Gray and Prisco, 1997). Increased high GBO activity with heightened directed attention (Hauck et al., 2007), etc.

It is feasible to regulate GBOs through their important roles in vision, hearing, attention, and other areas to achieve therapeutic goals, and certain results have already been achieved. For example, in the case of Alzheimer’s disease, researchers have found that optogenetic activation of PV interneurons at 40 Hz, which induces robust GBOs has also been shown to reduce amyloid load in 5XFAD mice (Adaikkan et al., 2019). Another study used light flickering to induce GBOs and found that gamma frequency light flickering enhanced the transport of amyloid precursor protein to the plasma membrane, reducing *β*-amyloid load in Alzheimer’s disease (Shen et al., 2022).

**Figure 1:**
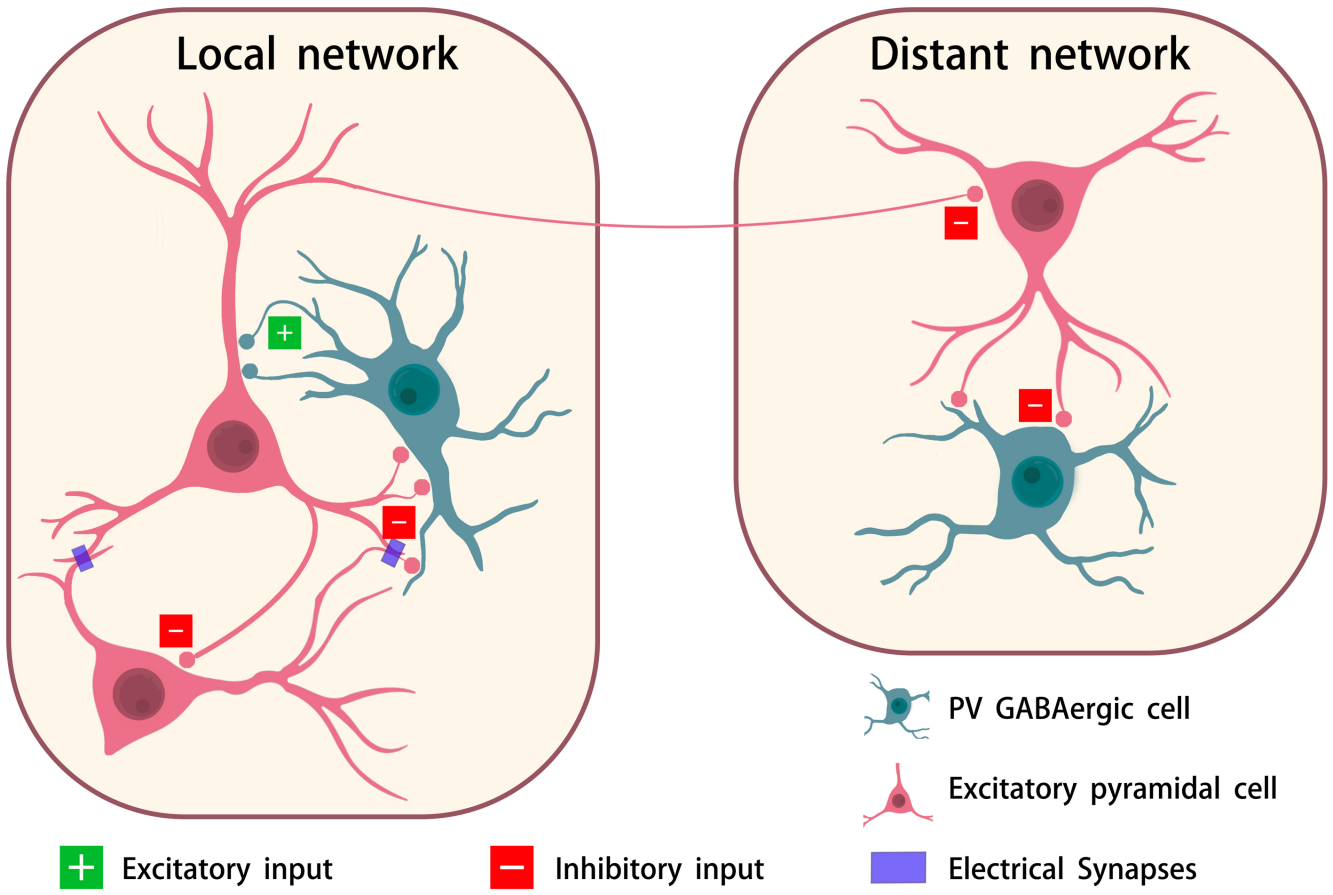
The primary mechanisms underlying the formation of GBO. Pyramidal neurons send excitatory signals through synapses to PV GABAergic neurons. Within PV neurons, electrical activity triggers hyperpolarization, converting these signals into inhibitory outputs. These inhibitory signals propagate back to the pyramidal neurons via synaptic feedback. Simultaneously, they spread to nearby pyramidal neurons through electrical and chemical synapses, amplifying local inhibition. Additionally, these signals project to distal brain regions for broader neural modulation. The figure was created with BioGDP.com (Jiang et al., 2025), and Scidraw.io.

GBOs are generated by the interaction between excitatory neurons and inhibitory neurons within a brain region (Ichim et al., 2024). PV neurons inhibit excitatory pyramidal cells through rapid firing and electrical synapses (Druga et al., 2023). GABA, as the primary inhibitory neurotransmitter, is released to activate GABAa receptors, suppressing the activity of excitatory neurons. This process confines neuronal firing within a narrow time window, ultimately giving rise to gamma rhythmicity (Milicevic et al., 2023). The core mechanism of GBO formation is shown in Figure 1.

These findings collectively suggest that interventions specifically targeting GBOs hold promise for alleviating pain symptoms and related disorders, underscoring the unique research value of GBOs compared to other neural oscillatory bands. Additionally, we highlight age-dependent variations in pain processing linked to cortical maturation. Studies have identified significant age-related differences in pain tolerance (Lautenbacher, 2012), with experimental data demonstrating that such tolerance positively correlates with prefrontal functional capacity. Notably, individuals with impaired prefrontal function exhibit increased GBOs characterized by a more dispersed spatial pattern. Furthermore, Noxious stimuli evoke gamma-frequency oscillations in both infants and adults. However, infants display markedly delayed gamma response onset (>500 ms post-stimulus), contrasting sharply with the immediate (<200 ms) and sustained (up to 2.5 s) GBOs observed in adults (Fabrizi et al., 2016; Price, 2000). Cortical functions in infants appear to engage wider cortical areas and evoke broader interactions across brain regions compared to adults. If the differences in GBOs across different cortical regions can be understood, optimal analgesic methods could be chosen during pain management.

This article reviews research on the cortical areas influenced by GBOs under pain conditions and the associated pain relief measures.

## 2 The cortical regions affected by pain-induced GBOs

**Figure 2:**
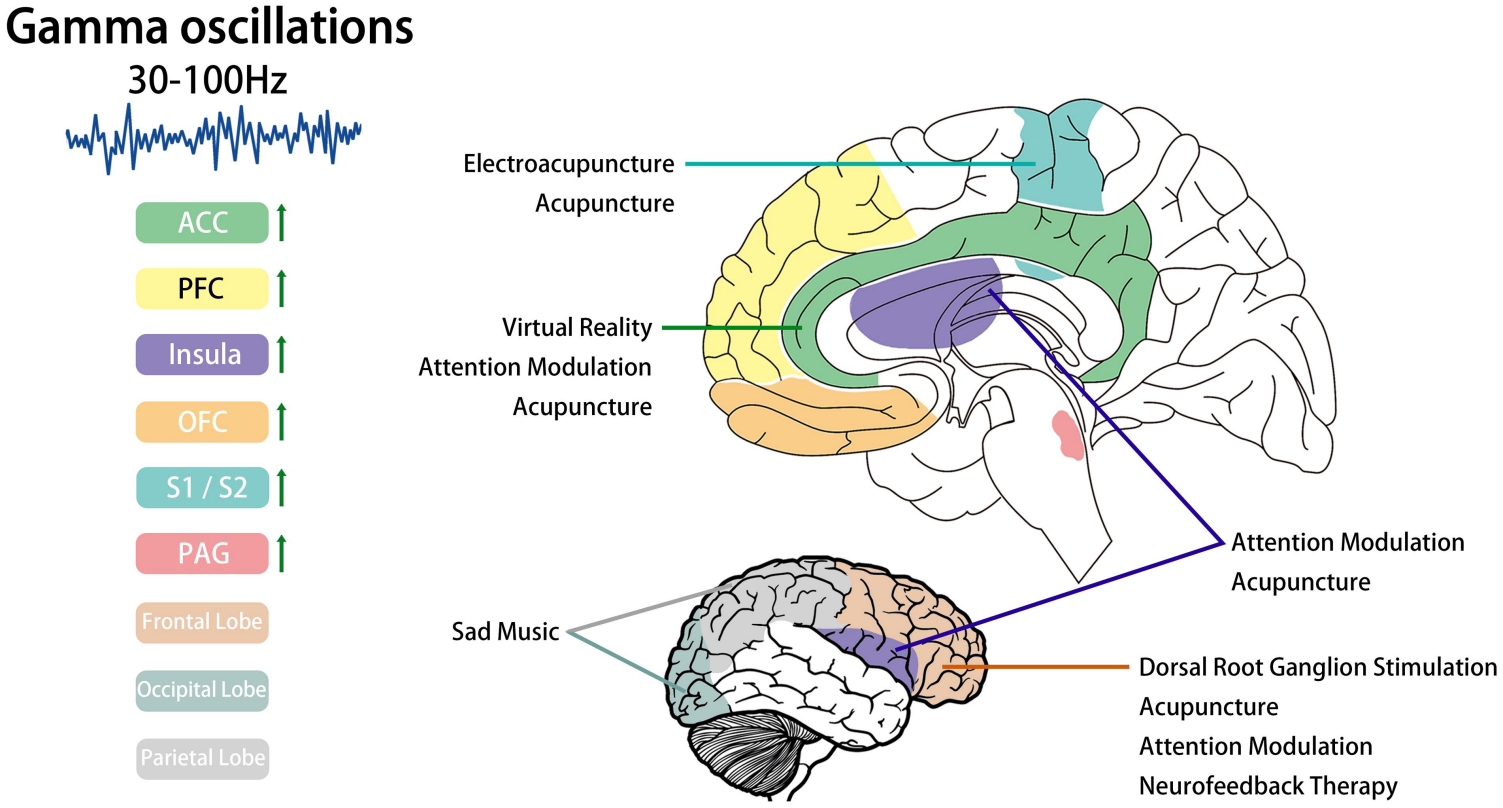
The left panel illustrates key cortical regions where GBOs occur, with areas exhibiting enhanced GBOs specifically highlighted (e.g. ACC, anterior cingulate cortex; PFC, prefrontal cortex; OFC, orbitofrontal cortex; S1, primary somatosensory cortices; S2, secondary somatosensory cortices; PAG, periaqueductal gray). The right panel provides a topographic mapping of these regions on a brain outline, further indicating cortical areas modulated by non-pharmacological interventions. Brain silhouette were sourced from https://scidraw.io/.

Many cortical regions are involved in processing information related to pain. The current pain processing pathways are the medial pathway, lateral pathway, and descending pathway. The medial pathway primarily involves the dorsal ACC and anterior insula, encoding the unpleasantness and distress of pain (Rainville et al., 1997; Price, 2000). The lateral pathway primarily involves the somatosensory cortex, handling the discrimination and sensation of pain (Flor et al., 1995; Bushnell et al., 2013). The descending pathway includes the pregenual ACC, periaqueductal gray, hypothalamus, and others (Kong et al., 2010), responsible for stress-mediated pain inhibition (Yilmaz et al., 2010) and placebo analgesia (Eippert et al., 2009). GBOs play a role or have been observed in multiple cortical regions, and due to the close relationship between the two, we review the cortical regions affected by pain-induced GBOs. The changes of GBOs in the relevant cerebral cortex are shown in Figure 2.

The primary somatosensory cortices (S1) and secondary somatosensory cortices (S2) predominantly process sensory discrimination, providing critical information regarding pain duration, intensity, and spatial localization. These regions also contribute significantly to the recognition and memorization of noxious events (Schnitzler and Ploner, 2000). Studies by Gross et al. have shown that selective nociceptive stimulation induces GBOs between 60 and 95 Hz in S1 (Gross et al., 2007). Yue et al. revealed robust temporal and phase coupling between superficial S1 layers and epidural GBOs (Yue et al., 2020). Notably, optogenetically induced GBOs in S1 enhance nociceptive sensitivity and evoke aversive avoidance behaviors (Tan et al., 2019).

S2 and posterior insular cortex likely constitute primary neural hubs that mediate attentional modulation of behavioral pain responses and subjective pain experience encoding (Lorenz and Garcia-Larrea, 2003). Studies have shown that pain-induced GBOs within S2 and insular regions are intrinsically modulated by directed attention (Lorenz and Garcia-Larrea, 2003). Betti et al. has specifically clarified this internal regulation that directly observing others’ pain elicits significantly enhanced GBO synchronization (33–90 Hz) in the observer’s sensorimotor system (Betti et al., 2009).

Schulz et al. established a positive correlation between pain intensity and GBOs during tonic pain. Furthermore, they demonstrated that GBOs in the medial PFC selectively encode the subjective perception of persistent pain (Schulz et al., 2015). Xuezhu Li et al. implanted electrodes in four pain related regions such as ACC, orbitofrontal cortex, and S1, and found that the power of the GBO was increased by applying harmful laser stimulation (Li et al., 2017).

ACC and insula are activated during pain anticipation or attentional engagement. Empathy—characterized by the capacity to understand others through shared intentions, emotions, and sensations (Preston and Waal, 2002)—engages pain empathy networks primarily involving the anterior insula and ACC (Hein and Singer, 2008). Ortega et al. discovered that during natural sleep in unanesthetized mice, somatosensory inputs from noxious versus innocuous stimuli differentially project to S1 and ACC, evoking complex transient and sustained responses across three frequency bands including GBOs (Sandoval Ortega et al., 2024). Furthermore, systemic hyperalgesia induces enhanced GBOs in the ACC (Kenefati et al., 2023).

Time-frequency analysis of EEG reveals that noxious stimuli elicit significant enhancement of GBOs in the insular cortexl (Liberati et al., 2018). Experimental evidence further confirms that activation of the anterior insula scales proportionally with magnitude ratings of both painful and visual stimuli (Baliki et al., 2009). Notably, patients with fibromyalgia exhibit persistent peak overactivation within the GBO across the left anterior insular cortex, primary motor cortex, and S1 (Lim et al., 2016).

## 3 Therapeutic regulation of GBOs

Current approaches to pain management encompass diverse therapeutic modalities, broadly categorized into pharmacological and non-pharmacological interventions. Mounting evidence recognizing the role of neural oscillations in neurological disorders which frequently manifest pain symptoms has spurred scientific interest in correlating specific pain phenotypes with GBOs to identify novel therapeutic targets.

### 3.1 Pharmacological modulators

Morphine has a strong analgesic effect and is suitable for various types of pain, so it is widely used (Wang et al., 2023). Whittington demonstrated that morphine activates *μ*-opioid receptors, leading to concentration-dependent modulation of network oscillations. At 20–50 μM concentrations, morphine induced a slight increase in population spike frequency during oscillations. Conversely, at higher concentrations (100–200 μM), it reduced the number of population spikes while increasing the prevalence of small amplitude high-frequency oscillations. They concluded that both morphine and *β*-endorphin impair GBOs within neuronal networks and their associated long-range synchrony (Whittington et al., 1998). Tramadol, another widely prescribed centrally acting opioid analgesic, elicits a marginal reduction in gamma-band power during wakefulness while enhancing GBOs during non-rapid eye movement sleep states (Koncz et al., 2021).

The endocannabinoid system plays a critical role in pain modulation. Delta-9-tetrahydrocannabinol has been demonstrated to increase GBO power in the anterior nucleus accumbens (NAc) while decreasing gamma power in the posterior shell region of the NAc. This bidirectional modulation correlates with its mechanism of inducing both rewarding and aversive effects via μ- and *κ*-opioid receptors, respectively (Norris et al., 2019).

The ablation and antagonism of N-methyl-D-aspartate (NMDA) receptors on PV neurons have been demonstrated to induce GBOs (Pinault, 2008; Korotkova et al., 2010). Ketamine, as an NMDA receptor antagonist, produces sedation, analgesia, and dissociation at low doses, while eliciting profound unconsciousness with antinociception at high doses (Adam et al., 2024). Notably, it exerts analgesic effects under both acute and chronic administration regimens (Noppers et al., 2010; Ye et al., 2018). Ketamine is currently used as a treatment for depression (Zanos and Gould, 2018), where pain constitutes a common comorbidity (Sheng et al., 2017).

Intriguingly, however, studies demonstrate that ketamine elicits GBOs in EEG recordings. The underlying mechanism involves ketamine’s disruption of the slow-unblock kinetics of NMDA receptor channels, which reduces interneuron activity and consequently induces disinhibition. This effect paradoxically contradicts conventional analgesic approaches that typically suppress GBOs (Slovik et al., 2017). Additional regulatory mechanisms may exist—for instance, research by Adam et al. reveals that ketamine disrupts and alters the excitatory-inhibitory balance, thereby modifying neuronal states. This discrepancy warrants further investigation (Adam et al., 2024).

Propofol, an intravenous anesthetic agent, induces loss of consciousness by enhancing GABAa receptor-mediated hyperpolarizing inhibition of pyramidal neurons (Brown et al., 2011). It has been thought to have analgesia enhancing properties due to activation of the GABAa receptor, a mechanism that may counteract the pronociceptive systems (Chidambaran et al., 2015). Propofol used commonly for the induction and maintenance of anesthesia, procedural, and critical care sedation in children (Chidambaran et al., 2015).

The functionality of PV interneurons is regulated by determinants of synaptic plasticity. For instance, SynCAM1 is specifically expressed in hippocampal PV neurons and participates in excitatory mossy fiber inputs from pyramidal neurons to PV interneurons in the hippocampus. Sevoflurane downregulates SynCAM1 expression, and the reduction in mossy fiber inputs impedes GBO generation (Zhao et al., 2023). Similarly, mefloquine downregulates electrical synapses composed of Connexin 36 in the ACC, mediating attenuated GBO and thereby alleviating neuropathic pain (Chen et al., 2016).

Lappaconitine, a potent analgesic extracted from the roots of natural Aconitum genus plants, demonstrates effective pain relief in its derivatives—Lappaconitine hydrobromide and Lappaconitine trifluoroacetate—on Sprague–Dawley rats subjected to noxious laser stimuli. Both derivatives significantly suppress nocifensive behaviors and reduce the amplitude of laser-evoked potentials, particularly in the GBO component. Compared with Lappaconitine hydrobromide, Lappaconitine trifluoroacetate exerts a more pronounced inhibitory effect on GBO magnitude and resting-state spectral power (Teng et al., 2021).

The anesthetic *α*-Chloralose enhances GABAa receptor function. Patch-clamp recordings demonstrated that α-Chloralose potentiates GABAergic leak currents and prolongs the decay constant of spontaneous inhibitory postsynaptic currents, thereby suppressing hippocampal GBO (Wang et al., 2008). It has been employed widely as an animal anesthetic in the laboratory setting (Holzgrefe et al., 1987).

Mitragyna speciosa (Kratom), employed as a traditional remedy for alleviating depression and pain, has gained increasing popularity in Europe and North America. Buckhalter’s experimental evaluation of kratom’s effects on neuronal oscillations and analgesia revealed that both high and low doses produced analgesic effects. Repeated administration of a low dose downregulated beta rhythms and elevated gamma power in the cingulate cortex, while repeated high-dose kratom selectively suppressed high-gamma power in the PFC and enhanced coherence of electrical activities across multiple brain regions (Buckhalter et al., 2021).

The effects of each drug on GBOs are shown in Table 2.

**Table 2:** The effects of each drug on GBOs.

Drug	Modulating effects
Morphine	Disrupts gamma oscillations in neuronal networks
Naloxone	Elevates gamma oscillations, enhancing awareness of pain worsening after opioid blockade.
Tramadol	Acute tramadol slightly reduces gamma power during wakefulness, while increases it during non-REM sleep
Delta-9-THC	Increases GBO power in anterior NAc, decreases gamma power in posterior NAc shell region
Ketamine	Increases cortical gamma power with power of GBOs
Propofo	Elevates gamma power in anterior/posterior cingulate cortex and enhances functional gamma connectivity between these regions
Sevoflurane	Reduces GBOs with decreased synCAM1 expression in PV interneurons and diminished PV phenotype
Mefloquine	Modulates GBOs and synaptic plasticity in anterior cingulate cortex
Lappaconitine	Significantly reduces laser-evoked potential amplitudes in GBO
α-Clorazepate	Suppresses hippocampal GBOs
Mitragyna speciosa	Repeated low-dose kratom administration suppresses beta/low-gamma power in cingulate cortex

### 3.2 Non-pharmacological interventions

Non-pharmacological approaches represent effective alternatives for pain alleviation. Compared to pharmacological interventions—particularly opioids—they significantly reduce addiction risks. In nations with stringent psychotropic drug regulations, these methods offer a safer therapeutic alternative. Research has further elucidated their mechanistic pathways associated with gamma rhythm.

Stimulation of the dorsal root ganglion is a neuromodulation intervention particularly ideal for alleviating localized chronic pain conditions. In a clinical study by Morgalla, nine patients diagnosed with chronic neuropathic pain underwent dorsal root ganglion stimulation therapy. The results demonstrated a significant reduction in numeric rating scale on the painful limb, accompanied by a marked decrease in resting-state gamma power within the 30–45 Hz range (Morgalla et al., 2024).

Deep brain stimulation (DBS), a neurosurgical intervention widely adopted for movement disorders such as Parkinson’s disease, has now been utilized as a therapeutic approach to alleviate refractory pain of diverse etiologies. Pereira demonstrated that DBS targeting the dorsal periaqueductal and periventricular gray reduces gamma power through increased release of endogenous opioids, correlating with significant pain relief. Administration of the opioid receptor antagonist naloxone alongside DBS antagonized the DBS-induced reduction in GBO indicating heightened perceptual sensitivity to pain exacerbation under opioid blockade (Pereira et al., 2013). Despite the existence of multiple anatomical targets beyond dorsal periaqueductal and periventricular gray—including cortical regions such as the anterior cingulate—a notable paucity of research persists in this domain (Boccard et al., 2015).

Research by Mark P. Jensen et al. revealed that transcranial direct current stimulation induced a small but statistically significant reduction in GBO. They proposed that this reduction mediates analgesic effects by attenuating or interrupting attentional processes or intra-cerebral communication. However, no significant linear association was identified between changes in GBO activity and alterations in pain intensity (Jensen et al., 2013).

Transcutaneous electrical nerve stimulation (TENS) is a noninvasive, cost-effective, and safe analgesic technique employed to alleviate both acute and chronic pain (Kara et al., 2010). High-frequency TENS can reduce enhanced GBO activity following induced tonic pain in healthy volunteers, demonstrating a decrease in total gamma power (Ebrahimian et al., 2018).

In a study by Hauck et al. investigating acupuncture’s effect on experimental pain induced by laser radiant heat stimulation, EEG revealed significantly greater attenuation of pain-related GBO in the bilateral PFC, S1, midcingulate cortex, and insula during acupuncture intervention. The authors propose that acupuncture may modulate pain through a shift in the interaction of different cortical sensory, limbic and executive networks with the default mode network and the autonomic nervous system (Hauck et al., 2017).

Interestingly, through the innovative modification of applying microcurrents between adjacent needles in traditional Chinese acupuncture, an emerging electroacupuncture therapy has been developed. This technique precisely integrates the principles of both acupuncture and TENS, thereby providing enhanced therapeutic efficacy. Consequently, clinical feedback from Chinese hospitals indicates that electroacupuncture is more favorably received compared to either modality used alone. Ding explored the relationship between electroacupuncture and acute postoperative pain, revealing that GBO alterations serve as biomarkers of pain modulation. Application of electroacupuncture corrects aberrant elevations in the amplitude of three oscillatory bands including gamma, thereby effectively alleviating painful hypersensitivity (Ding et al., 2023). Although the mechanism by which electroacupuncture modulates neural oscillations to produce analgesic effects remains to be fully elucidated, it has been extensively studied in the context of cerebrovascular accidents, Alzheimer’s disease, and pain disorders (Li et al., 2021). Considerable insights have been gained particularly regarding its modulation of neurotransmitters such as GABA, providing valuable implications for further research.

Rustamov investigated whether attentional processes facilitate pain inhibition in patients with irritable bowel syndrome. The experiment revealed that both heterotopic noxious counterstimulation and selective attention reduced pain-related high-gamma power in the sensorimotor cortex, ACC, and left dorsolateral (Rustamov et al., 2020).

Neurofeedback behavioral therapy, as a neuromodulation-based approach, serves as a long-term analgesic therapy (Moshkani Farahani et al., 2014). Research by Hamed et al. revealed that GBOs are modulated by neurofeedback, with enhancement in power observed across four neural oscillation bands—including GBOs—in the frontal regions (Hamed et al., 2022).

While the aforementioned physical therapies achieve analgesia by directly acting on pain-affected body parts or through conscious interventions, earlier discussions have noted that a series of sensory stimuli can also modulate neural oscillations. This suggests that leveraging auditory and visual signals may open new avenues for pain treatment. Virtual reality (VR) has emerged as a non-pharmacological adjuvant for pain management. Li demonstrated that distinct neural mechanisms, including gamma rhythms, underlie VR-induced analgesia (Li et al., 2023). Further observations revealed that the intensity of VR immersion positively correlates with pre-stimulus GBO enhancement, with significantly greater pain reduction during multisensory experiences. This is attributed to VR’s multisensory integration, which more effectively distracts attention compared to unimodal visual or auditory stimuli, thereby reducing pain intensity and unpleasantness perception. Notably, the experimental sequence—administering immersion prior to pain stimulation—deviates from real-world scenarios and may impact the validity of the conclusions.

Music can profoundly influence subjects’ emotional states and attentional focus, and is now frequently employed as an experimental stimulus in various analgesic studies (Dobek et al., 2014; Warth et al., 2018). Current research has partially elucidated its engagement with pain pathways (Garza-Villarreal et al., 2015). Guo demonstrated that sad music exhibits superior analgesic efficacy, attributed to enhanced beta-band oscillations and GBOs at O2 and P4 electrodes (Guo et al., 2020). However, the study has considerable limitations: the selection of simulated pain types was singular, personality factors significantly confounded the results, and the sample size of 40 participants was markedly inadequate. The cortical regions where GBOs are modulated by non-pharmacological interventions are shown in Figure 2.

The effects of each treatment on GBOs are shown in Table 3.

**Table 3:** The effects of each treatment on GBOs.

Intervention	Modulatory effect
Dorsal Root Ganglion Stimulation	Reduced resting-state gamma power
DBS	Increased endogenous opioid release and decreased gamma power
Transcranial direct current stimulation	A small but statistically significant reduction in GBO
TENS	Reduced enhanced gamma-band activity following tonic pain
Acupuncture	Reduced GBOs in bilateral PFC, S1, midcingulate cortex, and insula
Electroacupuncture	Attenuated amplitudes of three oscillations (including gamma), alleviated painful hypersensitivity, and restored gamma rhythm power
Attention Modulation	Selective attention reduced pain-related high-gamma power in sensorimotor cortex, ACC, and left dorsolateral PFC
Neurofeedback Therapy	Enhanced power across four bands (including gamma) in frontal regions
VR	Increased pre-stimulus spontaneous GBOs
Sad Music	Higher gamma-band amplitude in parietal regions correlates with sad emotion valence, while occipital gamma amplitude stems from selective attention

## 4 Research challenges and future directions

Pain constitutes an essential life experience that permeates human existence, with its perception representing a complex neurobiological phenomenon. Neural oscillations—rhythmic neuronal activities within the central nervous system—play a pivotal role in this process. GBOs ubiquitously distributed across cortical and subcortical regions are intrinsically linked to fundamental cognitive functions including attention, memory, and executive control. As synthesized in this review, GBOs demonstrate robust associations with pain pathophysiology, exemplified by the positive correlation between gamma power and subjective pain intensity. Critically, these oscillations serve as biomarkers for diverse pain conditions, such as chronic neuropathic pain and persistent back pain.

The therapeutic landscape targeting GBOs encompasses diverse modalities, ranging from pharmacological agents (e.g., ketamine, tramadol, and propofol) to non-pharmacological interventions (e.g., electroacupuncture, dorsal root ganglion stimulation, VR, and TENS). This diversity provides a framework to rationally harness GBOs in pain-prominent disorders, evolving beyond their role as biomarkers to serve as objective metrics for analgesic efficacy evaluation, and novel targets for analgesic development.

This review highlights the promising analgesic efficacy of both VR and music-based interventions, wherein GBOs play an integral role in their underlying neuromodulatory mechanisms. These approaches represent a paradigm shift in the therapeutic application of GBOs for pain management. While traditional non-pharmacological therapies predominantly rely on acupuncture and electrical stimulation, emerging strategies utilizing photo-acoustic stimuli to entrain GBOs have demonstrated considerable success. Current evidence suggests that physical signal-based neuromodulation targeting neural oscillations holds unprecedented potential for mechanistic exploration and clinical translation. This review synthesizes evidence on GBO-mediated analgesia across pharmacological and non-pharmacological therapies. Nevertheless, current research exhibits significant limitations, including: Insufficient standardization of experimental pain conditions when evaluating oscillatory responses to interventions. Methodological heterogeneity in quantifying GBOs (e.g., power versus amplitude metrics).

This systematic review has identified multiple pharmacologic and non-pharmacologic therapies that exert analgesic effects through GBO modulation. However, significant limitations persist in current GBO research. First, numerous studies simplistically attribute analgesia efficacy to gross GBO alterations, while overlooking critical inconsistencies in directional trends of waveform parameters—including spatial topography, amplitude, power spectral density, and peak frequency—across experimental paradigms. Although neural oscillations are classified by frequency bands with functionally distinct roles, GBOs fundamentally represent artificially defined rhythmic activities (30–100 Hz) that encode heterogeneous neural functions spanning pain subtypes and non-pain processes. Functionally distinct GBOs exhibit divergent patterns across these multidimensional attributes, which cannot be accurately discerned through human visual inspection. EEG, capturing direct neuroelectrical dynamics with high temporal resolution, remains the optimal modality for investigating oscillatory mechanisms. Integrating EEG data with AI-driven machine learning enables.

Systematic investigation of location- and phenotype-specific pain signatures. Extraction of discriminative waveform features. Development of diagnostic algorithms balancing specificity (>85%) and sensitivity (>90%). This approach facilitates mechanistic exploration beyond pain states to include emerging analgesics and neuromodulatory interventions. Quantifying intervention-induced gamma dynamics across cortical regions provides. Precision guidance for clinical treatment selection. Mechanistic biomarkers for novel analgesic development. Establishing an evidence-based framework for therapeutic innovation.

Second, a critical schism exists between pharmacological and non-pharmacological research paradigms. Pharmacological studies emphasize molecular pathways while neglecting neuroanatomical correlates, whereas non-pharmacological research focuses on local EEG dynamics without mechanistic depth. This disconnect precludes identification of shared mechanistic pathways for cross-modality analysis, confining efficacy assessments to macro-level outcomes—an approach that proves insufficient. In clinical practice, combined pharmacological and neuromodulatory therapies are frequently employed to enhance analgesic outcomes. However, the absence of comparative mechanistic data forces such combinations to rely predominantly on clinicians’ empirical judgment rather than evidence-based medical principles, often failing to yield optimal regimens. Compounding this challenge, the vast heterogeneity in pain phenotypes and underlying mechanisms necessitates mechanism-targeted matching between specific pain entities and therapeutic modalities, which constitutes a critical determinant for selecting first-line clinical strategies.

Finally, the prevalent limitation of small sample sizes merits critical attention. Given the methodological complexity of inducing targeted pain states in animal or human subjects prior to intervention, most studies are constrained to cohorts ranging from as few as 4 to several dozen participants. While such sample sizes may demonstrate proof-of-concept under current constraints, they remain inadequate for generating robust, statistically powered conclusions. To establish high-grade evidence per evidence-based medicine standards, large-scale multicenter randomized controlled trials are imperative. However, practical challenges arise: the non-life-threatening nature of most chronic pain conditions and patients’ preserved activities of daily living limit willingness for prolonged hospitalization. Consequently, standardized monitoring and intervention delivery prove difficult to implement. Large-scale longitudinal real-world studies represent a viable alternative pathway, leveraging digital phenotyping to capture ecological treatment responses across extended observation periods.

Furthermore, current research has not fully elucidated the causal relationship between GBOs and pain. Most literature focuses on the unidirectional causality where pain induces neural oscillations. However, emotions such as depression and empathic responses can also lead to somatic pain (Hein and Singer, 2008; Dudeney et al., 2024), with somatization in depression serving as a classic manifestation. This evidence suggests a bidirectional causality between the two phenomena.

In summary, while GBO research has established a rich foundation for pain therapeutics, these methodological limitations necessitate paradigm-shifting approaches.
